# A Case of Acute Coalescent Mastoiditis With Early Diagnosis of Lupus Anticoagulant-Hypoprothrombinemia Syndrome Prompting Immediate Surgical Drainage

**DOI:** 10.7759/cureus.84336

**Published:** 2025-05-18

**Authors:** Yota Saito, Masahiro Hasegawa, Kotaro Araki

**Affiliations:** 1 Department of Pediatrics, Okinawa Prefectural Miyako Hospital, Okinawa, JPN; 2 Department of General Pediatrics, Okinawa Prefectural Nanbu Medical Center and Children's Medical Center, Okinawa, JPN; 3 Department of Otorhinolaryngology, Okinawa Prefectural Nanbu Medical Center and Children's Medical Center, Okinawa, JPN

**Keywords:** acute coalescent mastoiditis, acute otitis media, fusobacterium nucleatum, lahps, lupus anticoagulant-hypoprothrombinemia syndrome, surgical drainage

## Abstract

Acute coalescent mastoiditis is a severe complication of acute otitis media characterized by bone destruction in the mastoid process and a risk of serious complications such as abscess formation and intracranial spread. Prompt surgical management is often required, especially in cases with evidence of disease progression. We report the case of a four-year-old girl who developed acute coalescent mastoiditis complicated by transient lupus anticoagulant-hypoprothrombinemia syndrome (LAHPS). Despite initial antibiotic treatment, the patient’s symptoms worsened, and imaging revealed bone erosion in the mastoid region. Laboratory tests showed significantly prolonged activated partial thromboplastin time, raising concern for bleeding during surgery. A cross-mixing study and bleeding assessment revealed low bleeding risk, supporting the decision to proceed with surgical drainage and mastoidectomy. The procedure was completed safely, and cultures identified *Streptococcus intermedius* and *Fusobacterium nucleatum* as the causative organisms. This case highlights the importance of rapid diagnosis, careful evaluation of bleeding risk, and timely surgical intervention in managing coalescent mastoiditis, especially when complicated by coagulation abnormalities like LAHPS.

## Introduction

Acute mastoiditis, a well-known complication of acute otitis media, is characterized by physical findings such as swelling, redness behind the ear, and auricular protrusion. Recent reports have suggested that conservative treatment alone may be sufficient in some cases. However, if inflammation spreads to the mastoid sinus, it may progress to coalescent mastoiditis, increasing the risk of bone destruction, abscess formation, and intracranial complications. Without appropriate surgical intervention, complications such as hearing loss may occur. Thus, surgical indications should be considered carefully in patients with acute mastoiditis [1-3]. Lupus anticoagulant-hypoprothrombinemia syndrome (LAHPS) is a rare autoimmune disorder characterized by the presence of lupus anticoagulant antibodies combined with acquired hypoprothrombinemia, which can lead to an increased risk of bleeding complications during surgical procedures. We recently treated a case in which mastoidectomy was performed at an appropriate time to address coalescent mastoiditis, despite the presence of a prolonged activated partial thromboplastin time (APTT), following careful evaluation of bleeding risk after rapid diagnosis of LAHPS.

## Case presentation

The patient was a four-year-old girl. Seven days prior to admission to our center, she visited a local doctor complaining of fever, swelling, and pain in the back of her left ear, for which she was administered amoxicillin for five days. However, she was later referred to our hospital because her swelling and pain worsened despite treatment.

**Figure 1:**
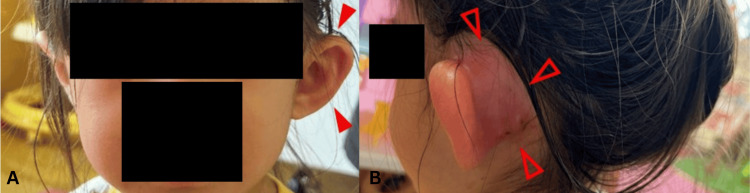
Physical findings showing the (A) protruding auricle and (B) localized redness and swelling behind the left ear

On physical examination, she was alert, her pulse was 136 beats per minute and regular, her respiration was 22 beats per minute, and her temperature was 38 degrees centigrade. Our initial examination revealed localized redness and swelling in the posterior left ear, and we noted that the auricle was "protruding," as if being pushed forward (Figure 1). She had no cavities. Otoscopic examinations demonstrated purulent effusion behind a bulging tympanic membrane. Blood tests showed an elevated inflammatory response with a white blood cell count of 13,520/µL, C-reactive protein level of 6.97 mg/dL, and significantly prolonged APTT of 141 s. The patient's prothrombin time values fell within the reference range (Table 1). She had no underlying diseases and was in generally healthy condition, and she was not taking any medication that could prolong APTT.

**Table 1:** Laboratory findings on admission

Laboratory findings	Value	Reference range
White blood cell count	13.52	3.3-8.6 x 10^3 /μL
Neutrophils	52.5	47.0-61.0%
Lymphocytes	37.1	25.0-45.0%
Monocytes	8.5	4.0-7.0%
Hemoglobin	10.1	11.6-14.8 g/dL
Hematocrit	31.5	35.1-44.4%
Platelet count	53.9	13-35 x 10^4/μL
Blood urea nitrogen	6	8-20 mg/dL
Serum creatinine	0.25	0.46-0.79 mg/dL
Aspartate transaminase	34	13-30 IU/L
Alanine transaminase	10	7-23 IU/L
C-reactive protein	6.97	0.00-0.14 mg/dL
Prothrombin time (sec)	12.7	10-13 sec
Prothrombin time (%)	83	70-130%
Activated partial thromboplastin time	141	25-34 sec

**Figure 2:**
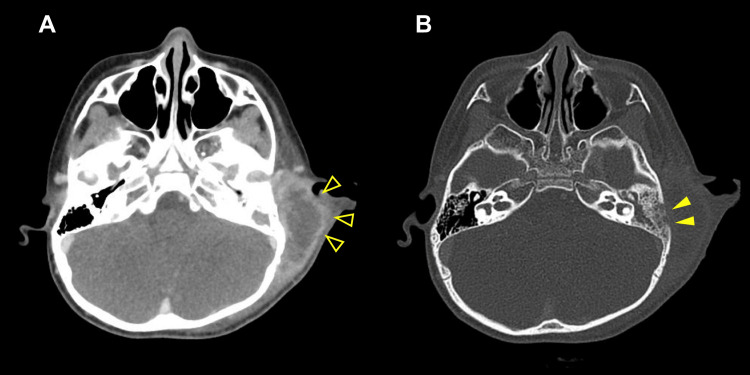
Contrast-enhanced CT In the left auricular bone protrusion, (A) loss of air in the auricular cavity, a low-density area suggesting abscess formation, and (B) bone dissolution were observed. CT: computed tomography

On contrast-enhanced computed tomography, the patient's left mastoid process showed air loss in the mastoid cavity, a low-absorption area suggesting the formation of an abscess, and bone dissolution. These findings led to a diagnosis of mastoiditis-associated bone destruction (Figure 2).

There were concerns regarding the possibility of progression to intracranial complications and hearing loss, meaning that emergency surgery was indicated. However, because of the risk of bleeding caused by the patient's coagulation abnormalities, only abscess drainage via puncture aspiration was performed.

**Figure 3:**
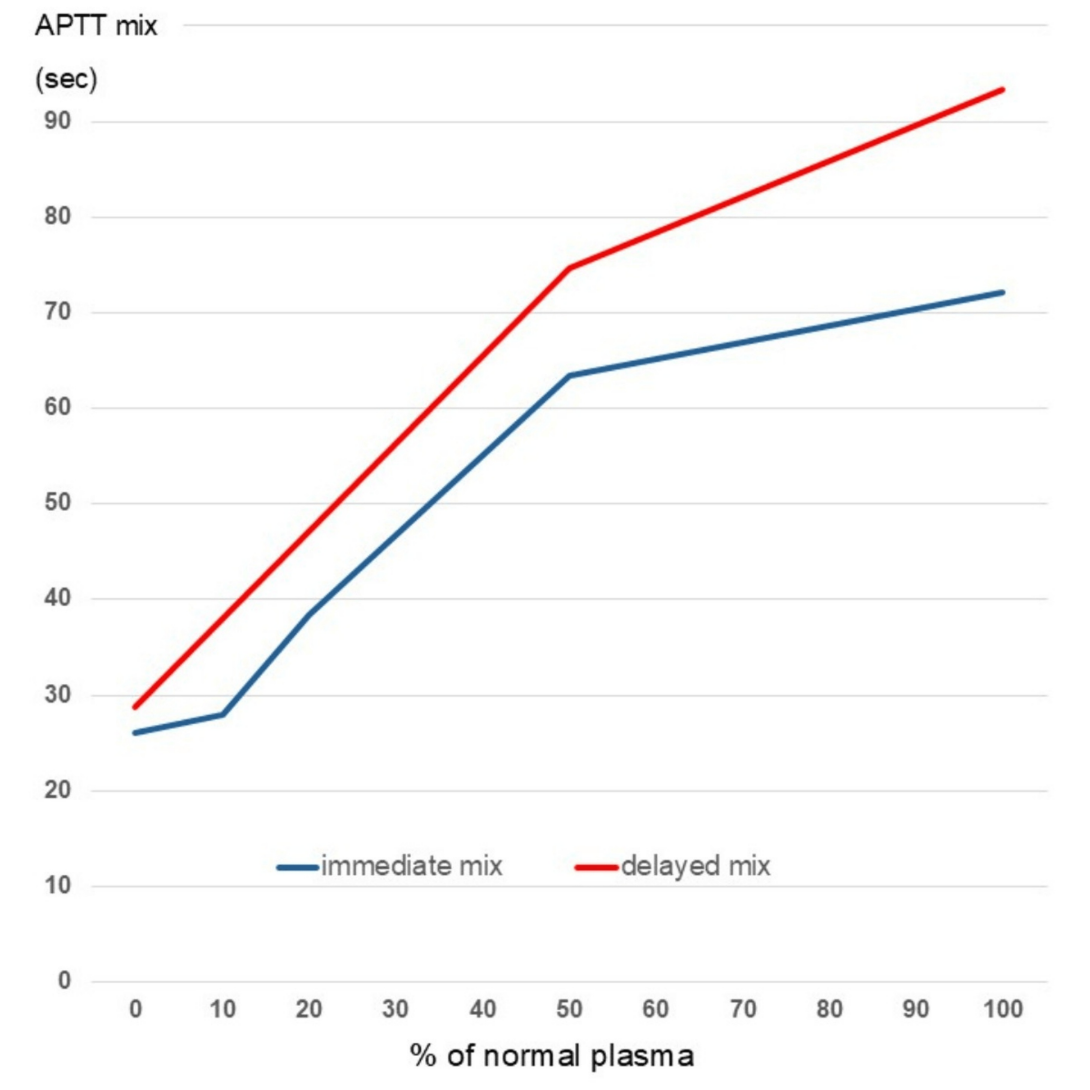
Cross-mixing test The corrected curve showed an upward convexity for both immediate and delayed types. APTT: activated partial thromboplastin time

Considering the possibility of *Staphylococcus aureus* and/or *Streptococcus pneumoniae* infection, intravenous administration of cefotaxime and vancomycin was initiated. On the second day of hospitalization, her fever persisted, and swelling had not improved. A cross-mixing test was performed to measure her corrected APTT after mixing her plasma samples with normal plasma. The corrected curve showed an upward convexity for both immediate and delayed types, and the presence of an inhibitor such as lupus anticoagulant was suspected (Figure 3).

The patient had a bleeding score of 0 on the International Society on Thrombosis and Haemostasis-Bleeding Assessment Tool (ISTH-BAT) test, indicating low bleeding risk [4]. Thus, otolaryngological incision and drainage, as well as mastoidectomy, were performed, and a tympanostomy tube was inserted. Steroid therapy for LAHPS was not initiated, as the bleeding risk was deemed low, the syndrome was considered a transient form secondary to infection, and emergency surgical intervention was required. Intraoperative bleeding was minimal, and the postoperative course was uneventful.

**Figure 4:**
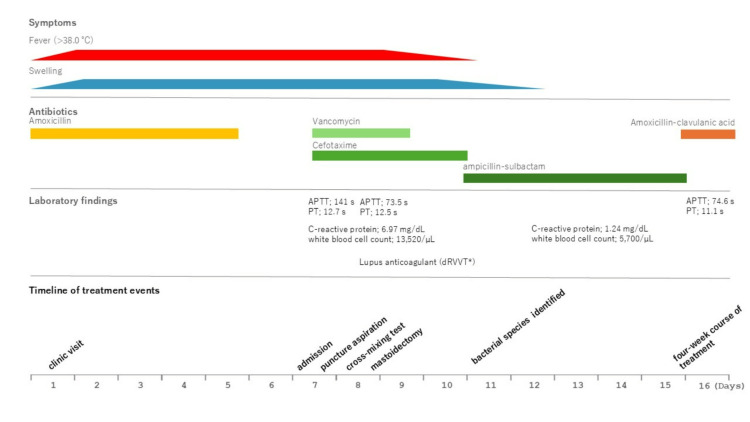
Clinical course from symptom onset to hospital discharge APTT: activated partial thromboplastin time, PT: prothrombin time, dRVVT: diluted Russell’s viper venom time

*Streptococcus intermedius* and *Fusobacterium nucleatum* were detected in the puncture specimen following aerobic and anaerobic cultures. Anaerobic culture yielded more than 100 colonies of *Fusobacterium nucleatum*, which supports its pathogenic role in this case; therefore, a four-week course of amoxicillin/clavulanic acid was administered and completed. After confirming the resolution of postauricular swelling, she was discharged on hospital day 11 (Figure 4). Lupus anticoagulant positivity was confirmed using the dilute Russell's viper venom time test (Table 2).

**Table 2:** Additional tests on the seventh day after onset dRVVT: diluted Russell’s viper venom time, IgG: immunoglobulin G

Laboratory findings	Value	Reference range
Lupus anticoagulant (dRVVT*)	1.7	<1.1
Anti-cardiolipin IgG	<4 U/mL​	<10 U/mL
Anti-cardiolipin-β2 glycoprotein 1 complex antibody	≤1.2 U/mL	<3.5 U/mL
Antinuclear antibody	×160	
C3	200 mg/dL​	80-165 mg/dL
C4	31 mg/dL​	14-42 mg/dL
Factor II	100%	75-135%
Factor VIII	101%	60-150%
Factor IX	113%	70-130%
Factor XI	126%	75-145%
Factor XII	99%	50-150%
Factor VIII inhibitor	Negative	Negative
Factor IX inhibitor	Negative	Negative

The LA test was negative four months later, and APTT was normalized. No auditory sequelae were observed at the follow-up evaluation after discharge. One year has passed since symptom onset, and no systemic lupus erythematosus (SLE) or other autoimmune diseases have developed, nor has LAHPS recurred.

## Discussion

This was a pediatric case in which surgical treatment for mastoiditis was performed at the appropriate time because the condition was promptly diagnosed as transient LAHPS associated with an infection. Even though surgical intervention was initially delayed due to a possible differential diagnosis of hemophilia, to our knowledge, there are no previous reports of coalescent mastoiditis complicated by LAHPS in a pediatric patient.

LAHPS is a rare blood disorder that has been reported in a small number of pediatric cases. This acquired coagulopathy is often associated with SLE but can also be precipitated by certain infections or drugs. The symptoms of LAHPS vary from asymptomatic to potentially life-threatening bleeding. Typical laboratory findings include extended APTT, extended prothrombin time (not always present), positivity for LA, and prothrombin factor II deficiency [5-7].

Mulliez et al. reported that 55% of LAHPS cases (n=74) were related to autoimmune diseases, and infectious diseases triggered 33%. Most of those cases occurred after acute gastroenteritis or upper respiratory tract infection, with adenovirus being the most common viral infection. Among them, 66 (89%) experienced bleeding symptoms [8].

Tian et al. reported that ISTH-BAT bleeding scores were lower in the group with infection-associated conditions (median, 4; interquartile range, 2-4; p=0.03) than in those with autoimmune-related conditions (median, 6; interquartile range, 4-7), among 70 pediatric cases of LAHPS [9]. In cases caused by infection, symptoms are often mild and transient, with significant bleeding occurring in approximately 17% of cases. The bleeding symptoms tended to be more severe in patients with prothrombin activity levels <10% [9]. In the present case, the cause was considered to be infection, and a bleeding tendency was not observed in the patient, whose prothrombin activity was 83%.

Various viral infections have been reported to cause transient LAHPS; however, case reports of LAHPS caused by bacterial infections are rare.

In the present case, *Streptococcus intermedius* and *Fusobacterium nucleatum* were detected in the patient’s drainage culture, leading us to believe that these caused the patient’s mastoiditis. However, the otitis media that preceded her mastoiditis was most likely caused primarily by a viral infection that preceded the others and caused the patient’s LAHPS.

In this case, we could not confirm whether our patient had a decrease in factor II. We believe this is because the test was performed too long after infection onset. However, her APTT was confirmed to be prolonged, and she tested positive for LA, indicating a high probability of LAHPS. Furthermore, evaluations using the cross-mixing test and the patient’s bleeding score remained useful for determining an appropriate treatment.

A simple mastoidectomy is the most reliable and effective surgical treatment for acute mastoiditis. However, recent reports have suggested certain criteria that may warrant avoiding this invasive procedure [3,10-12]. According to Trigolet et al., mastoidectomy is recommended in cases with intracranial complications (e.g., subperiosteal abscess) [13]. Zanetti et al. reported the following as indications for mastoidectomy: exteriorized mastoid abscess, intracranial complications, cholesteatoma, purulent otorrhea, and resistance to topical and systemic antibiotics lasting ≥ 2 weeks [11]. In our case, owing to abnormal coagulation test results, we considered conservative treatment. However, bone destruction had already progressed, and the patient was diagnosed with coalescent mastoiditis. Because of the significant risk of progression to intracranial complications such as subdural abscess, we determined that early drainage was warranted and thus performed a mastoidectomy.

Acute coalescent mastoiditis is essentially a suppurative complication of acute otitis media and, if left untreated, can lead to severe sequelae, including subperiosteal abscess, meningitis, or intracranial abscess. Mansour et al. emphasize that early diagnosis and appropriate surgical intervention are critical to preventing these potentially life-threatening complications. Despite advances in diagnostic imaging and antimicrobial therapy, intracranial complications have been reported in up to 23% of cases when surgical treatment is delayed. Therefore, timely mastoidectomy remains an essential component of management when radiologic evidence of coalescence or signs of clinical deterioration are present [14].

## Conclusions

The early diagnosis of LAHPS through cross-mixing tests and a thorough bleeding risk assessment was integral to the clinical decision-making process. This methodical approach facilitated the precise determination of the optimal timing for surgical intervention, thereby minimizing the risk of hemorrhagic complications. Moreover, integrating diagnostic results with comprehensive clinical evaluation ensured that the surgical management of mastoiditis was conducted safely and effectively. This case underscores the critical importance of a structured, multidisciplinary strategy in managing coagulopathic patients presenting with complex infectious conditions, ultimately contributing to improved clinical outcomes.
